# Photodynamic inactivation of drug-resistant bacteria isolated from diabetic foot ulcers

**Published:** 2011-03

**Authors:** N Kashef, G Esmaeeli Djavid, M Siroosy, A Taghi Khani, F Hesami Zokai, M Fateh

**Affiliations:** 1 Department of Microbiology, School of Biology, College of Science, University of Tehran, Tehran, Iran.; 2 Iranian Center for Medical Lasers (ICML), Academic Center for Education, Culture and Research (ACECR), Tehran, Iran.

**Keywords:** Photodynamic inactivation, drug-resistant bacteria, wound infections

## Abstract

**Background and Objectives:**

Due to the extensive use of antibiotics, the spread of drug-resistant bacteria is one of the most worrisome threats to public health. One strategy that can be used to overcome potential shortcomings might be the inactivation of these organisms by photodynamic therapy. In this study, we have investigated whether drug-resistant wound-associated organisms (*Staphylococcus aureus*, *Staphylococcus epidermidis* and *Escherichia coli)* are sensitive to lethal photosensitization using the dye methylene blue coupled with laser light of 660 nm.

**Materials and Methods:**

Effect of photosensitizer concentration (25, 50, 100 µg/ml) and laser light dose (27.3, 54.6 and 109.2 J/cm^2^) on lethal photosensitization was investigated.

**Results:**

All species were susceptible to killing by photodynamic inactivation. The bactericidal effect was not dependent on the concentration of methylene blue but it was dependent on the light dose. Methylene blue photosensitization using red laser light (109.2 J/cm^2^) was able to achieve reductions of 99.03% and 98.95% in the viable counts of *S. aureus* and *S. epidermidis* (using starting concentrations of 10^4^–10^5^ CFU/ml). Eradication of 92.23% were obtained for *E. coli* (initial concentration 10^4^–10^5^ CFU/ml) photosensitized by the red light (109.2 J/cm^2^).

**Conclusion:**

These findings imply that MB in combination with red light may be an effective means of eradicating drug- resistant bacteria from wounds.

## INTRODUCTION

The increasing resistance of pathogenic micro- organisms against antimicrobial agents has led to the search for new treatments for localized infections. A potential alternative may be photodynamic therapy (PDT), which is based on the interaction of visible light and a photosensitizer agent, which under photo- activation generates short-lived cytotoxic species *in situ* (1). By photodynamic therapy of microorganisms, both antibiotic-sensitive and -resistant strains can be successfully inactivated (2–4). In addition, repeated photosensitization of bacterial cells does not induce a selection of resistant strains (5).

Superficial wound infections are potentially suitable for treatment by PDT because of the ready accessibility of these wounds for both topical delivery of the photosensitizer and light. The eradication of wound-infecting bacteria using photosensitization has been reported in the literature (6–9).

There are some studies considering the applica-tion of methylene blue (MB) solution for the treat-ment of tumor tissues and non-cancerous diseases (10–13). Also, several studies of photodynamic action of MB on pathogenic bacteria have been performed (4, 14, 15). MB easily crosses bacterial cell walls. Because of its cationic charge, it binds easily to the negative charge of the lipopolysaccharides of Gram-negative bacteria (16). Gram-positive bacteria have only capsular material and peptidoglycan outside of the cytoplasmic membrane, enabling MB to cross it easily as well (17). However, reports of its effectiveness against drug-resistant bacteria are limited. In this study, we have investigated whether drug-resistant wound-associated organisms are sensitive to lethal photosensitization using the dye MB coupled with laser light of 660 nm.

## MATERIALS AND METHODS

**Bacterial strains and culture conditions**. Two Gram-positive organisms were used in this study; *Staphylococcus aureus* and *Staphylococcus epidermidis*. In addition, the Gram-negative bacterium *Escherichia coli* were used. All three organisms were isolated from foot ulcer infections of diabetic patients. Antimicrobial susceptibility pattern of each isolate was done by the standard disk diffusion method according to CLSI recommendations (18). *S. aureus* was resistant to 11 antibiotics including amoxicillin- clavulanate (20/10 µg), ceftazidime (30 µg), oxacillin (1 µg), piperacillin (100 µg), cephalexin (30 µg), imipenem (10 µg), ciprofloxacin (5 µg), clindamycin (2 µg), erythromycin (15 µg), gentamicin (10 µg), and amikacin (30 µg). *S. epidermidis* was resistant to ceftazidime, oxacillin, piperacillin, erythromycin, co-trimoxazole (1.25/23.75 µg), doxycycline (30 µg), gentamicin, and amikacin. *E. coli* was resistant to amoxicillin-clavulanate, ceftazidime, piperacillin, cephalexin, imipenem, ciprofloxacin, levofloxacin, norfloxacin, co-trimoxazole, doxycycline, and gentamicin.

Organisms were maintained by weekly subculture on nutrient agar (Merck). All three strains were grown aerobically in Nutrient Agar plates at 37°C for 18-24 h. Then a suspension of each organism was prepared in sterile phosphate buffered saline (PBS) (pH=7.4) to a concentration of 10^4^-10^5^ CFU/ml.

**Fig. 1:**
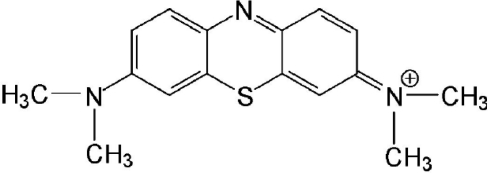
Molecular structure of methylene blue

**Photosensitiser (S) and light source**. Methylene blue (MB) was purchased from Sigma, UK (Fig. 1). Methylene blue solution was prepared fresh for each experiment in sterile PBS (pH=7.4), filter- sterilized and kept in the dark. A 35 mW diode Laser (Lasotronic – UK) emitting light with a wavelength of 660 nm was used for irradiation. For experimental purposes, the distance of the laser probe to the plate surface was adjusted to give fluence rate of 91 mW/ cm^2^.

**Effect of photosensitizer concentration on lethal photosensitization**. Aliquots (0.1 ml) of a suspension of *S. aureus*, *S. epidermidis*, and *E. coli* (containing approximately 104-105 CFU/ml) in sterile PBS were transferred into a 96-well plate and an equal volume of MB in PBS was added to each well to give final concentrations of 25-100 µg/ml.

After addition of the MB ranging from 25 µg/ml to 100 µg/ml, the wells were left in the dark for 30 minutes (pre-irradiation time) and then exposed to a measured dose of laser light at a fluence rate of 0.091 W/cm^2^.

In this system, an exposure of 10 minutes corresponded to a light dose of 54.6 J/cm2. Each experimental condition was tested 5 times and each experiment was carried out on four occasions. The conditions tested were: 1) controls which contained neither MB nor received irradiation (L-S-), 2) incubation with MB in the dark (L- S+), 3) irradiation in the absence of MB (L+S-) and 4) the test which was irradiated in the presence of MB (L+S+). The plates were kept covered during the illumination in order to maintain the sterility of the culture.

To enumerate the surviving bacteria, serial 10-fold dilutions were plated on nutrient agar.

**Effect of laser light dose on lethal photo- sensitization**. The effect of laser light dose on bacterial killing was investigated by varying the exposure time whilst the distance from the light source remained constant. The bacterial suspensions were prepared as described above. A photosensitizer concentration of 50 µg/ml was used for photosensitizing the organisms. Survival was determined after 5, 10, and 20 minutes irradiation at a fluence rate of 0.091 W/cm2, corresponding to light doses of 27.3, 54.6 and 109.2 J/cm^2^, respectively.

**Statistics**. Values were expressed as log means±concentration of the MB and using a light dose of standard deviation. Comparisons between means of groups were analyzed using the One-Way ANOVA test. P<0.05 was considered statistically significant.

## RESULTS

**Fig. 2:**
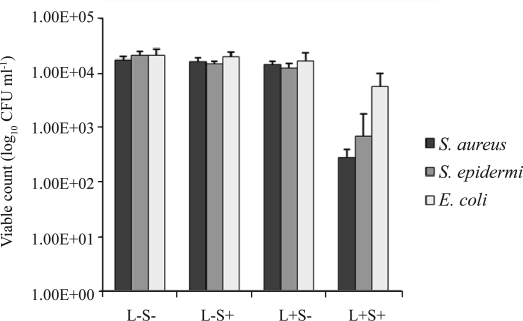
Lethal photosensitization of organisms with 25 µg/ml MB. An equal volume of either 50 µg/ml MB (L-S+ and L+S+) or PBS (L-S- and L+S-) was added to each bacterial suspension, samples were left for 30 minutes in the dark and then irradiated at a fluence rate of 0.091 W/ cm^2^ with a light dose of 54.6 J/cm^2^ from a red laser light (660 nm) (L+S- and L+S+) or kept in the dark (L-S+ and L-S-).

**Methylene blue is an effective photosensitizer of drug-resistant wound-infecting organisms**. When *S. aureus* was treated with different concentrations of MB and exposed to 54.6 J/cm^2^ of red light, a significant reduction in the viable count was achieved even with the minimum concentration of MB. For instance, when 25 µg/ml of MB was used there was a significant (P<0.001) reduction in the viable count of the suspension which contained 1.8×10^4^ CFU/ml corresponding to 98.41% efficacy. Suspensions of *S. aureus* treated with MB but not irradiated (L-S+) or those irradiated in the absence of MB (L+S-) did not show a reduction in the viable count (Fig. 2).

When *S. epidermidis* was treated with different concentrations of MB and exposed to 54.6 J/cm2 of red light, a significant reduction in the viable count was achieved even with the lowest concentration of MB. Treatment with 25 µg/ml of MB gave approximately a 96.83% reduction (p<0.001) in the viable count. This amounted to a kill of 2.08×104 CFU/ml. Bacteria treated with MB but not irradiated (L-S+), or those not treated with MB but irradiated with red light (L+S-) did not show a significant reduction in viability (Fig. 2).

When *E. coli* was treated with different concentrations of MB and exposed to 54.6 J/cm2 of red light, a significant reduction in the viable count was achieved even with the minimum concentration of MB albeit not as great as that achieved in Gram- positive organisms. For instance, when 25 µg/ml of MB was used there was a significant (P=0.023) reduction in the viable count of the suspension. This equated to killing of approximately 7.84×10^3^ CFU/ ml corresponds to 57.65% efficacy. Irradiation of *E. coli* with red light in the absence of MB or treatment with MB alone did not result in a significant reduction in the viability of this organism (Fig. 2).

**Fig. 3:**
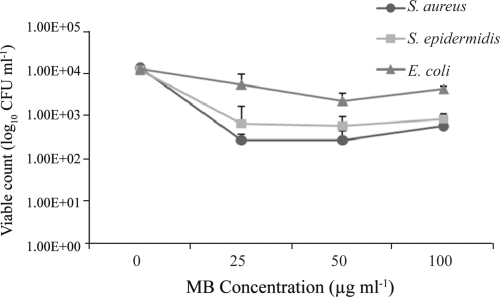
Lethal photosensitization of organisms with 25, 50, and 100 µg/ml MB. An equal volume of the appropriate MB concentration (L+S+) was added to each bacterial suspension, samples were left for 30 minutes in the dark and then irradiated at a fluence rate of 0.091 W/ cm^2^ with light dose of 54.6 J/cm^2^ from a red laser light (660 nm).

**The effect of various dye concentrations**. The bactericidal effect in all species was not dependent on the MB concentration. Fig. 3 shows the log unit reduction in the viable count in the three microorganisms when treated with different 54.6 J/cm2 at a fluence rate of 0.091 W/cm2.

**Fig. 4:**
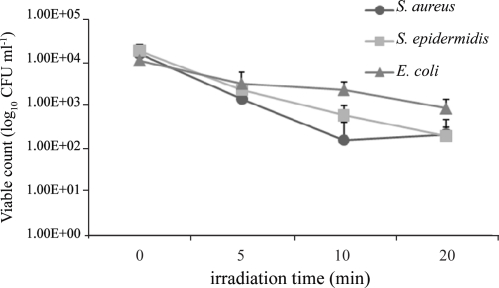
Lethal photosensitization of organisms with 50 µg/ ml MB. An equal volume of 100 µg/ ml MB (L-S+ and L+S+) or PBS (L-S- and L+S-) was added to each bacterial suspension. Samples were left in the dark for 30 minutes and then (L+S- and L+S+) irradiated at a fluence rate of 0.091 W/cm^2^ for 5, 10 or 20 minutes with red laser light (660 nm), corresponding to light doses of 27.3, 54.6, or 109.2 J/cm^2^ respectively. Samples L-S+ and L-S- were kept in the dark.

**The effect of light dose**.During irradiation, the bactericidal effect was dependent on the light dose delivered (Fig. 4). Significant (P<0.001) reductions of 91.25%, 99.03%, and 99.03% in the viable count of *S. aureus* were achieved using exposure times of 5, 10 and 20 minutes, respectively. Significant (P<0.001) reductions in the viable count of *S. epidermidis* were 87.79%, 96.84%, and 98.95% using light doses of 5, 10 and 20 minutes, respectively. In the case of *E. coli*, lethal photosensitization using exposure times of 5, 10 and 20 minutes achieved significant (P<0.001) reductions of 71.02%, 79.15% and 92.23% in the viable count, respectively. However, in the absence of MB, irradiation of three organisms did not result in significant kills (p>0.05).

## DISCUSSION

Methylene blue is a widely known histological dye that has been in use for many years (19). It belongs to the phenothiazinium class of compounds (20). MB has shown *in vivo* activity against several types of tumors when locally injected and illuminated with red laser light (21, 22). Photosensitization reactions induced by MB excitation are known to cause damage to nucleic acids, proteins and lipids (19). To understand its effect on drug-resistant bacteria, we evaluated the efficacy of antibacterial photodynamic inactivation on drug-resistant *S. aureus*, *S. epidermidis*, and *E. coli*, using MB as the photosensitizer, followed by red light irradiation. MB was an effective photosensitizer of these wound-infecting organisms, although the reduction in the viable count of *E. coli* was not as great as that achieved in Gram-positive organisms. Different susceptibilities of the Gram-negative and Gram-positive organisms to lethal photosensitization in this study are probably due to differences in cell wall structures. Gram-negative bacteria have an outer membrane that may reduce the uptake of reactive oxygen species by the bacterium. In contrast, Gram-positive bacteria have a porous outer layer of peptidoglycan which is a less effective permeability barrier (23).

The concept of disinfecting burns and wounds using a noninvasive and localized strategy such as PDT with limited damage to the host tissue is well documented in the literature (24–26). Lambrechts *et al*. Achieved 3.6 or 4.8 log units reduction in the viability of *S*. The data obtained in this study have shown that *aureus* using 635 nm light with a light dose of 0.6 or 1.5 J/cm2 and 1.56 µM 5-phenyl-10,15,20-tris (N-methyl-4-pyridyl) porphyrin chloride (26). Taking into account the variation in experimental design, we achieved a significant reduction of 99.03% in the viability of *S. aureus* using 50 µg/ml MB and a light dose of 54.6 J/cm2 from a red light source (660 nm). In another study, Orenstein *et al*. used a mixture of deuteroporphyrin and hemin which successfully disinfected burns infected with *S. aureus* even in the dark without illumination (8). In contrast, the MB used in the current study had no dark toxicity against the organisms tested. According to Peloi *et al*. report, the percentage growth rate of *S. aureus* and *E. coli* decreased as the MB concentration increased (14-70 µM and 35-140 µM, respectively) (1). However, the bactericidal effect in all species was not dependent on the MB concentration in our study. It seems that photosensitization reactions induced by MB excitation would not increase as the MB concentration increased from 25 to 100 µg/ml (67-268 µM).

Laser light alone was not able to exert a bactericidal effect. However, the results of Omar *et al*. study showed that irradiation of *P. aeruginosa* with 411 J/cm2 laser light at a wavelength of 808 nm and using an irradiance rate of 1.37 W/cm2 resulted in a significant inhibition of bacterial growth (27). Lethal- photosensitization was dependent on exposure time to the laser light. This finding is supported by other investigations (1, 27) _._

The data obtained in this study have shown that significant kills of *S. aureus* and *S. epidermidis* can be achieved using a low concentration of the MB of 25 µg/ml and a light dose of 54.6 J/cm2. The gramnegative organism, *E. coli*, appeared to be less susceptible as higher light doses were needed to achieve substantial kills.

In summary, the results of the present study suggest that MB in combination with red laser light is a promising candidate for the photodynamic therapy of burn and wound infections caused by drug-resistant bacteria. Use of this approach would reduce the requirements for systemic antibiotics in the management of skin infections and thereby help to reduce the emergence of antibiotic resistance. Although the results of these in vitro studies are promising, in vivo studies are needed to determine whether considerable bacterial kills can be obtained in a wound model.
